# Work-Nonwork boundaries in academia: A problematizing review

**DOI:** 10.12688/openreseurope.18573.2

**Published:** 2025-02-05

**Authors:** Jūratė Čingienė, Aleksandra Batuchina

**Affiliations:** 1 SMK University of Applied Social Sciences, Klaipėda, Klaipėda County, Lithuania

**Keywords:** Work-nonwork boundaries, Academic work, Problematizing review

## Abstract

The interplay between work and non-work in academic settings has been the subject of extensive research, particularly in relation to work-life balance and work-nonwork conflict. However, much of this literature has tended to overlook the specific dynamics of work-nonwork boundaries. Moreover, while prior research has explored general patterns of conflict and balance, it has not sufficiently addressed the unique pressures that academics face, such as high autonomy, irregular working hours, and competing demands. This review critically examines how the specific nature of academic work shapes the boundaries between work and non-work, advancing the conversation beyond traditional approaches. The central research question guiding this review is: How do the aspects of academic work shape the blurring of work-nonwork boundaries? Through a problematizing approach, this review relies on 41 articles to broaden and enhance our understanding of the boundary challenges academics encounter. Findings reveal that blurred work-nonwork boundaries in academia are driven by work-life demand overload, work-family conflicts, and a lack of organizational support, compounded by digitalisation and neoliberal practices. Heightened managerialism, careerism, and precarity exacerbate the blurring of these boundaries, affecting academics' well-being and identity work. By addressing these gaps, this review offers a nuanced understanding of how academics construct, navigate, and negotiate boundaries within a complex environment shaped by these pressures. The review challenges the limitations of conventional approaches to work-nonwork interface advocating for a more context-sensitive, experiential perspective.

## Plain language summary

This review examines how academic work affects the boundaries between professional and personal life. Although there is extensive research on work-life balance and work-nonwork conflict, much of it does not address the work-nonwork boundary nuances and the specific challenges that academics face, such as long, irregular hours, high levels of independence, and increasing demands in teaching, research, and service. These factors often blur the lines between work and personal time.

The review analyzes 41 studies and highlights several key issues. For instance, academic work frequently spills into personal time due to constant connectivity and pressure to always be available. Additionally, pressures related to career advancement and institutional demands make it even more challenging for academics to maintain clear boundaries. As a result, many experience stress, mental health issues, and decreased satisfaction in both work and personal life.

The review emphasizes the need for a more nuanced understanding of work-life boundaries in academia. It critiques traditional views that see boundary negotiation simply as a problem to be solved. Instead, it advocates for recognizing the fluid and complex nature of these boundaries, shaped by factors like gender roles, neoliberal pressures, and the demands of modern academic work. By addressing these unique challenges, the review calls for healthier, more sustainable work-life boundaries and increased support from academic institutions to better support academics in navigating their professional and personal lives.

## Introduction

The relationship between work and nonwork in academia has been extensively studied, particularly with regard to concepts such as work-life balance ( Beddoes & Pawley, 2014; Kinman & Jones, 2008; Rosa, 2022) and work-nonwork conflict ( Beigi *et al.*, 2018), along with their antecedents ( Hogan *et al.*, 2014). While these studies offer valuable insights into the intersection of work and nonwork, they often overlook the distinct issue of boundaries - a growing challenge in contemporary academia. Academic work is increasingly characterized by blurred boundaries between professional and personal life, contributing to significant mental health and psychological well-being concerns ( Docka-Filipek & Stone, 2021; Edwards *et al.*, 2021).

Although the concepts of work-life balance and work-family conflict are important, they primarily focus on managing multiple roles or mitigating work-nonwork role conflict. However, they fail to fully capture how work and nonwork boundaries become increasingly permeable, especially in the academic profession, where constant connectivity, irregular working hours, and high levels of autonomy exacerbate boundary blurring ( Cohen *et al.*, 2009). The pressure to balance multiple professional roles—such as research, teaching, and service—coupled with the growing demands for productivity and excellence, has intensified these challenges, contributing to stress and mental health struggles ( Griffin, 2022; Hogan *et al.*, 2014).

Academia shares challenges with other knowledge-intensive occupations ( Alvesson, 2004), including autonomy, non-standard work hours, and role multiplicity. Still, the specific dynamics of academic work render the negotiation of work-nonwork boundaries particularly complex. While these characteristics are widely recognised as complicating the work-nonwork interface ( Kelliher & Anderson, 2010; Reissner *et al.*, 2021), the literature - aside from a few notable exceptions ( Cohen *et al.*, 2009; Izak *et al.*, 2023) - often overlook their specific influence on the construction and negotiation of these boundaries within academic settings. Work-life balance discussions typically focus on how individuals manage competing personal and professional demands ( Rosa, 2022), while research on work-nonwork conflict primarily emphasizes role overload ( Beigi *et al.*, 2018), assuming a clear demarcation between work and personal life. However, these perspectives inadequately address the blurred and dynamic nature of boundaries in academia, where constant role overlap and professional pressures result in their frequent dissolution ( Izak *et al.*, 2023).

Moreover, work-nonwork boundaries extend beyond the mere achievement of balance or avoidance of conflict; they involve the intricate process of defining where work ends and personal life begins, encompassing dimensions such as mental engagement, physical actions, technology use, spatial arrangements, and social interactions ( Languilaire, 2009). In academia, where the expectation to be continuously available and productive is pervasive, the blurring of these boundaries exacerbates mental health challenges ( Docka-Filipek & Stone, 2021). As in the healthcare field, where boundary violations have been closely linked to burnout ( Rapp *et al.*, 2021), academics may benefit from employing boundary work tactics to redefine and protect their work-nonwork boundaries, potentially reducing burnout risks.

In reviewing the existing literature on work-nonwork boundaries, traditional approaches (e.g., Allen *et al.*, 2012; Beigi *et al.*, 2019; Byron, 2005; Casper *et al.*, 2007; Casper *et al.*, 2018) often aggregate and synthesize existing knowledge without critically examining the specific work contexts where these boundaries are negotiated. This approach overlooks the nuanced complexities of academic work, where the boundaries between professional and personal life are constantly renegotiated, with significant implications for mental health and well-being ( Docka-Filipek & Stone, 2021; Edwards *et al.*, 2021). To address these complexities, it is essential to move beyond traditional reviews and develop a more critical understanding of work-nonwork boundaries in academia.

This review aims to address these gaps by providing a critical examination of work-nonwork boundaries within academia, with a particular emphasis on how the work context influences the construction and negotiation of these boundaries. The central research question guiding this review is: *How does the nature of academic work contribute to the blurring of work-nonwork boundaries in academia*? By employing a problematizing approach, this review seeks to move beyond traditional frameworks of work-life balance and conflict, offering a nuanced and contextually specific understanding of boundary dynamics within an academic setting.

## Theoretical framing

### Defining work-nonwork

The term "work-family" is often used in the work-nonwork interface literature to describe the interplay between work and family responsibilities, encompassing activities that may not be confined to specific physical locations. However, this can inadvertently exclude individuals whose significant nonwork activities extend beyond family-related responsibilities ( Özbilgin *et al.*, 2011). To address this limitation, this review adopts a broader perspective, considering a range of nonwork activities and social contexts that contribute to the work-nonwork interface. In this article, the term "work-nonwork" is preferred over "work-life." The latter suggests a separation between work and life, which can be misleading, as work is a significant and often dominant part of many people's lives ( Lewis & Cooper, 2005). The term "work-nonwork" here more accurately reflects the integration of work with other aspects of life, acknowledging that the boundaries between them are not always distinct.

### Work-nonwork boundary theory

Boundary theory ( Ashforth *et al.*, 2000; Kreiner *et al.*, 2009; Nippert-Eng, 1996) examines how individuals define and manage the boundaries between work and personal life, focusing on the segmentation-integration continuum. At one end of this continuum, segmentation refers to maintaining a clear separation between work and personal life, while at the other end, integration describes the blending of these domains. This perspective goes beyond traditional concepts such as work-family conflict, defined as “a form of interrole conflict in which role pressures from work and family domains are mutually incompatible” ( Greenhaus & Beutell, 1985, p. 77). Unlike work-life balance, which Carlson *et al.* (2009) define as an individual’s ability to meet the demands of both work and other life roles, boundary theory recognises that the boundaries between work and personal life are not always distinct. Instead, it highlights the fluidity of modern work contexts, where individuals must continuously negotiate overlapping roles, challenging the notion of a clear-cut division between work and nonwork domains.

Research on work-nonwork boundaries reveals various types or dimensions: *temporal boundaries, physical boundaries, mental boundaries, digital boundaries, spatial boundaries, and social boundaries* ( Clark, 2000; Languilaire, 2009; Nippert-Eng, 1996). *Temporal boundaries* refer to the division of time between work and personal life. *Physical boundaries* pertain to the physical separation between work and personal spaces. *Mental boundaries* involve the cognitive separation between work and personal life. This dimension addresses how easily individuals can disengage mentally from work-related thoughts and concerns. *Digital boundaries* relate to the use of technology and its impact on separating work from personal life. *Spatial boundaries* involve the preferences and practices related to where work is conducted. This dimension explores the distinction between different physical locations used for work and personal activities. *Social boundaries* refer to the nature of relationships between colleagues and the extent to which professional interactions extend into personal life. *Micro transitions* involve the small, often routine, actions taken to move from work to personal time, such as physically returning home after work. These transitions help in psychologically and physically shifting from work to a personal mode ( Ashforth *et al.*, 2000; Languilaire, 2009). Each of these types -temporal, physical, mental, digital, spatial, social boundaries, and micro transitions—shows different nuances in how individuals may experience boundaries.

## Methods

We adopted a problematizing review approach to critically reassess existing understandings of the work-nonwork boundaries in academia and to offer insights into this distinctive context. Drawing on Alvesson and Sandberg's (2020) problematizing review principles, this review emphasized reflexivity, selective reading, and problematization rather than merely accumulating knowledge. We used reflexivity as a central principle to the review process, encouraging a critical examination of established conventions and assumptions within the work-nonwork literature. This involved interpreting texts critically, questioning dominant paradigms, and exploring alternative perspectives. By doing so, the review aimed to challenge prevailing logic and propose new interpretations. Alvesson and Sandberg’s (2020) selective reading strategy enabled a broader yet focused view of the field, incorporating diverse perspectives to enhance critical reflection and counteract narrow viewpoints. Rather than aggregating data, the review prioritized problematization—defining the scope of the literature, identifying underlying assumptions, and questioning problematic elements. This approach sought to uncover new insights by reflecting on these assumptions and exploring alternative views. Additionally, the review adhered to the "less is more" principle, conducting interpretative readings of selected texts to evaluate their construction of phenomena critically. The aim was not to discredit existing knowledge but to offer constructive critiques that expand conventional understandings and facilitate fresh perspectives in the field.

The philosophical foundation of this review was grounded in critical realism and contextualism. Contextualism posits that multiple interpretations of reality can coexist, and a particular account is not rendered invalid by the existence of a conflicting one; however, some interpretations may be more compelling or valuable than others ( Madill *et al.*, 2000). Furthermore, we acknowledge that the researcher’s values and practices inevitably shape the research process and its outcomes ( Braun & Clarke, 2006; Braun & Clarke, 2022).

### Article selection process: a three-level approach

**Figure 1.**
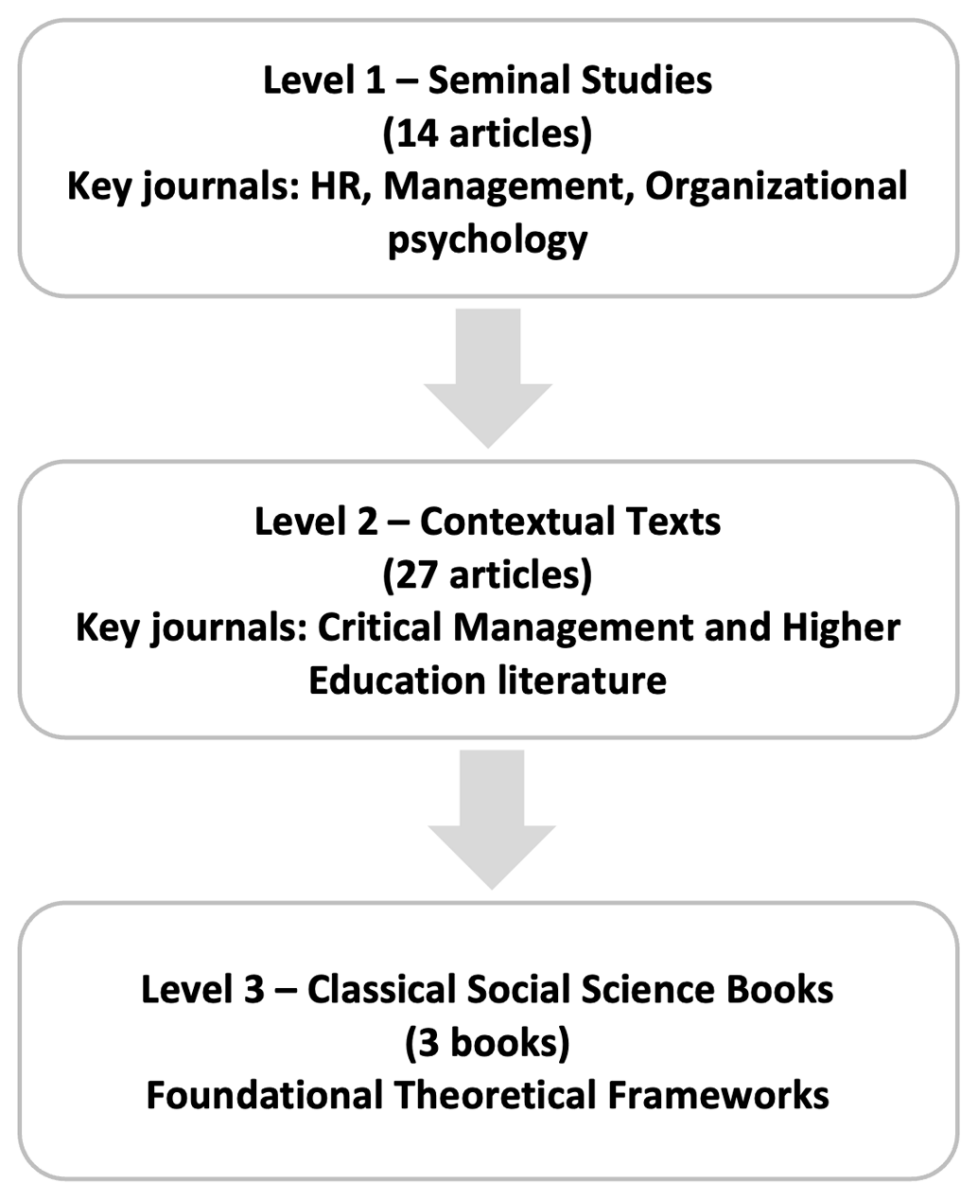
Article Selection Process: A Three-Level Approach.

Our article selection process follows a comprehensive, three-tiered approach broadly guided by Alvesson and Sandberg (2020) to ensure a robust and representative review of the literature concerning work-nonwork boundaries, within academic settings (See Figure 1). This approach incorporated seminal works, relevant contemporary studies, and influential classics with indirect relevance.

At the first level, we established a foundational understanding of the phenomenon by engaging in an in-depth review of 14 studies that specifically address the work-nonwork interface within the academic context. This process involved a selective and iterative reading approach to identify key insights and emerging patterns. To ensure both a historical perspective and a comprehensive understanding of evolving dynamics of work-nonwork boundaries in academia, we included studies published between 2009 and 2016, alongside more recent research from 2017 to 2023. For instance, Ylijoki’s (2013) seminal work was among the first to examine work-nonwork boundaries within the "high-speed university" context, highlighting the increasingly accelerated pace of academic work. Kossek *et al.* (2021) offered valuable insights into how academic women in STEM across 202 universities navigated the disruption of work-nonwork boundaries during the COVID-19 pandemic, reflecting significant shifts in boundary management in response to recent challenges.

Additionally, we incorporated several review articles (e.g., Beigi *et al.*, 2018; Rosa, 2022) to provide a broad and synthesized understanding of the phenomenon across diverse studies. This combination of historical and contemporary works enabled us to capture the complexity and shifting nature of work-nonwork boundaries in academia. Articles were sourced from human resource management, management, organizational psychology, and higher education journals, such as *Human Resource Development Review, Journal of Management Inquiry, Journal of Applied Psychology, Management Learning* and *Gender, Work and Organization*. After identifying key articles, we conducted cross-reference searches to find additional relevant works. Search phrases included: ‘work-nonwork,’ ‘work-life,’ ‘personal life,’ ‘work-family,’ ‘boundaries,’ ‘conflict,’ ‘balance,’ ‘academic,’ and ‘researcher’ (see Table 1).

**Table 1.** Level 1 Readings.

Author(s)	Year	Title	Publisher/Journal
Adisa, T. A., Antonacopoulou, E., Beauregard, T. A., Dickmann, M., & Adekoya, O. D.	2022	Exploring the Impact of COVID-19 on Employees’ Boundary Management and Work–Life Balance	*British Journal of Management*
Beigi, M., Shirmohammadi, M., & Stewart, J.	2018	“Flexible Work Arrangements and Work–Family Conflict: A Metasynthesis of Qualitative Studies among Academics”	*Human Resource Development Review*
Beddoes, K., & Pawley, A. L.	2014	‘Different people have different priorities’: Work–family balance, gender, and the discourse of choice	*Studies in Higher Education*
Branch, J., Chapman, M., & Gomez, M.	2021	Investigating the interplay between institutional, spousal, parental and personal demands in tenure track faculty everyday life	*Community, Work & Family*
Bozzon, R., Murgia, A., Poggio, B., & Rapetti, E.	2017	Work–life interferences in the early stages of academic careers: The case of precarious researchers in Italy	*European Educational Research Journal*
Cohen, L., Duberley, J., & Musson, G.	2009	“Work—Life Balance? An Autoethnographic Exploration of Everyday Home—Work Dynamics”	*Journal of Management Inquiry*
Hogan, V., Hogan, M., Hodgins, M., Kinman, G., & Bunting, B.	2014	“An Examination of Gender Differences in the Impact of Individual and Organizational Factors on Work Hours, Work-Life Conflict and Psychological Strain in Academics”	*The Irish Journal of Psychology*
Izak, M., Shortt, H., & Case, P.	2023	“Learning to Inhabit the Liquid Liminal World of Work: An Auto-ethnographic Visual Study of Work-Life Boundary Transitions”	*Management Learning*
Johnston, K., Tanwar, J., Pasamar, S., Van Laar, D., & Bamber Jones, A.	2022	“Blurring Boundaries: Work-Life Balance and Unbounded Work in Academia. The Role of Flexibility, Organisational Support and Gender”	*Labour and Industry*
Kossek, E. E., Dumas, T. L., Piszczek, M. M., & Allen, T. D.	2021	“Pushing the Boundaries: A Qualitative Study of How STEM Women Adapted to Disrupted Work–Nonwork Boundaries During the COVID-19 Pandemic”	*Journal of Applied Psychology*
Rosa, R.	2022	The trouble with ‘work–life balance’ in neoliberal academia: a systematic and critical review	*Journal of Gender Studies*
Smith, C., & Ulus, E.	2020	Who cares for academics? We need to talk about emotional well-being including what we avoid and intellectualise through macro-discourses	*Organization*
Toffoletti, K., & Starr, K.	2016	Women academics and work–life balance: Gendered discourses of work and care	*Gender, Work and Organization*
Ylijoki, O.	2013	“Boundary-work between Work and Life in the High-Speed University”	*Studies in Higher Education*

At the second level, we expanded our scope by incorporating 27 key texts from related areas, such as critical management and higher education literature, to provide critical contextual insights (see Table 2 in the extended data repository). These texts focused on institutional contexts and academics' experiences, contributing to a more nuanced understanding of the review’s research question. Articles from journals such as the *Academy of Management Learning & Education* and *Organization Studies, Journal of Organizational Change Management and Organizations* were included. The key texts were carefully selected to provide critical contextual insights, drawing on perspectives from adjacent fields such as critical management and higher education literature. Although these studies did not specifically focus on work-nonwork boundaries, they were included to enrich understanding of the broader academic work context.

At the third level, we selected three seminal social science texts (see Table 3) to provide foundational and theoretical depth to our understanding of work-nonwork boundaries in academia. One of these texts directly addresses the negotiation of work-nonwork boundaries ( Nippert-Eng, 1996), offering a critical framework for examining boundary dynamics. The other two works ( Alvesson, 2004; Alvesson *et al.*, 2017) were included for their broader contributions to the theory of knowledge work and the academic environment, which are central to understanding the complexities of academic work. These texts were chosen not only for their conceptual relevance but also for their ability to inform the current academic context by addressing the evolving nature of work-nonwork boundaries, especially within knowledge-intensive fields. While acknowledging other important works, these texts were prioritized for their theoretical richness and their potential to deepen our analysis of boundary management in academia.

**Table 3.** Level 3: Readings.

Author(s)	Year	Title	Publisher/Journal
Alvesson, M.	2004	Knowledge Work and Knowledge-intensive Firms	Oxford University Press
Alvesson, M., Gabriel, Y., & Paulsen, R.	2017	Return to Meaning: A Social Science with Something to Say	Oxford University Press
Nipper-Eng, C. E.	1996	Home and Work: Negotiating Boundaries through Everyday Life	The University of Chicago Press

## Review process

Three researchers engaged in a systematic process of reading and re-reading the selected texts, documenting assumptions about the phenomenon, exploring alternative viewpoints, and identifying new perspectives. Each scholar independently identified key themes, which were then collaboratively discussed within the context of current academic realities, addressing both explicit and implicit dimensions. Reflexivity was central to this process, encouraging critical reflection on how both the researchers’ subjectivities and assumptions embedded in the literature influenced the interpretation and integration of findings.

This reflexive and collaborative approach facilitated evaluating the coherence of synthesized insights while appreciating divergent perspectives and was essential for achieving a rich and nuanced understanding of the work-nonwork interface in academia. The combination of independent and collaborative analysis allowed for the uncovering of hidden patterns, contradictions, and novel insights. Reflexivity ensured critical engagement with the literature and the researchers’ interpretations, enabling a layered, context-sensitive exploration of the phenomenon that acknowledged the researcher’s subjectivity ( Braun & Clarke, 2006; Braun & Clarke, 2022).

## Findings

This section presents the findings from our review of work-nonwork boundaries in academia discussing how the nature of academic work shapes the blurring of work-nonwork boundaries.

### Individual struggles with blurred and fluid work-nonwork boundaries

A recurring theme in the literature we reviewed is the ongoing struggles that academics face due to blurred temporal boundaries between professional and personal life. These struggles are exacerbated by everyday multiple work-life role demands ( Branch *et al.*, 2021; Cohen *et al.*, 2009) and work-family conflicts ( Hogan *et al.*, 2014; Pasamar *et al.*, 2020). Several studies have explored the experience of boundarylessness, examining how the lack of separation between work and personal life is navigated by academics ( Cohen *et al.*, 2009; Izak *et al.*, 2023). For instance, Izak *et al.* (2023) highlight the fluid and indeterminate nature of work-nonwork boundaries in academia. The non-traditional hours, self-directed tasks, and integration of personal intellectual pursuits with professional responsibilities inherent in academic work blur these boundaries. The constant negotiation of these boundaries is a fundamental aspect of the modern academic’s experience, characterized by "liquid liminality" ( Izak *et al.*, 2023; p.215). This concept refers to the process by which the distinction between work and nonwork is removed and continuously re-created through intersubjective sensemaking and symbolic mastery of spaces and objects that hold personal meaning for the individual.

Some research has specifically focused on the unique challenges faced by female academics ( Toffoletti & Starr, 2016), particularly those balancing their roles in academia with early motherhood ( Izak *et al.*, 2023; Lupu, 2021). For example, Lupu's (2021) autoethnography explores how personal experiences, such as pregnancy and maternity, challenge the "ideal academic" norms. Drawing on Bourdieu's concept of “illusio”, Lupu reflects on how her experiences of childbirth provided insights into resisting the entrenched culture of long working hours and personal sacrifice in academia. Her work contributes to a growing body of research considering how feminine, embodied experiences offer alternative perspectives and forms of resistance to dominant discourses on what constitutes the "ideal academic" (p.1899) (e.g., Docka-Filipek & Stone, 2021; Toffoletti & Starr, 2016).

### Dissolved boundaries during COVID-19

The COVID-19 pandemic significantly altered the nature of academic work, with teaching and research activities abruptly transitioning to home environments ( Adisa *et al.*, 2022; Docka-Filipek & Stone, 2021; Kossek *et al.*, 2021). This shift redefined work-nonwork boundaries for many academics, as working from home (WFH) became mandatory, and all members shared household spaces for work, study, and daily life ( Adisa *et al.*, 2022).

A study by Adisa *et al.* (2022) offers insights into how UK academics experienced these changes during the first lockdown. Their multi-method qualitative research revealed that while WFH is typically seen as a flexible arrangement, its compulsory implementation during the pandemic diminished this perceived flexibility. Academics reported a lack of instrumental and emotional support, such as reduced access to childcare and social interactions, along with increased workloads and employer surveillance. These challenges led to blurred boundaries between work and personal life, particularly for those who previously preferred to maintain a clear distinction between the two. In response, many academics employed strategies like ‘micro boundaries’ and time-based techniques to create ‘controlled integration.’

Exploring the gendered dimensions of academic work, Docka-Filipek & Stone (2021) investigated the impact of gender disparities in academic labour on faculty mental health during the initial COVID-19 lockdown. Surveying 345 faculty members, their research highlighted that women faced heightened risks of depression and anxiety, driven by increased teaching loads, caregiving responsibilities, and financial concerns. Crucially, these mental health risks were not solely linked to these external pressures but also to the often-overlooked service burdens within academia and the home. This underscores the broader issue of gendered care work and academic precarity, posing significant challenges to women’s well-being and career advancement during the pandemic. The COVID-19 pandemic has worsened existing gender disparities in academia, particularly concerning work boundaries, responsibilities, and workloads. According to Thébaud *et al.* (2024), women in academia experienced a more significant reduction in work hours compared to their male counterparts, largely due to increased caregiving responsibilities. This change adversely affected women's research productivity, mental health, career progression, and opportunities. Thébaud *et al.* (2024) emphasize that the issue lies not only in the heightened caregiving expectations women faced on average, but also in the inadequacy of university policies to support those caregivers who were most strained. Similarly, Górska *et al.* (2021) noted that the pandemic intensified the gendered nature of academic work, with women shouldering a disproportionate share of domestic duties. This imbalance created significant challenges in managing professional boundaries and maintaining productivity.

Building on these findings, Kossek *et al.* (2021) examined how women in STEM navigated disrupted work-nonwork boundaries during the pandemic. Their research showed that women employed a variety of strategies, such as concealing or revealing their nonwork roles (e.g., adjusting webcam angles to hide personal spaces) and making sacrifices in both work (e.g., stepping back from major projects) and nonwork roles (e.g., reducing personal activities). Notably, structural support—such as flexible work arrangements—and social support at work were critical in helping women manage these challenges effectively.

### Academic work context shaping the blurred boundaries

The individual struggles with blurred boundaries, as discussed earlier, highlight the tensions inherent in academics' daily experiences. A growing body of research examines how the academic work environment intensifies work-nonwork challenges, with key factors including flexible yet unbounded work arrangements ( Beigi *et al.*, 2018; Johnston *et al.*, 2022), gendered academic roles ( Berheide *et al.*, 2022; Docka-Filipek & Stone, 2021), neoliberal management practices ( Rosa, 2022; Ylijoki, 2013), and the global demands of academia ( Haley *et al.*, 2024). However, specific aspects of the academic context shaping these boundaries remain insufficiently explored. To address this gap, we integrate insights from critical management and educational studies with work-nonwork interface research, offering a more nuanced understanding of how flexible work, gendered expectations, managerialism, and global demands shape boundary dynamics in academia.

***Flexible work and unbounded work in academia*.** While flexible work arrangements are often seen as beneficial for academics, they can complicate boundary management, exacerbating work-nonwork conflicts and diminishing work-life balance ( Beigi *et al.*, 2018; Johnston *et al.*, 2022). Beigi *et al.* (2018), in their review of 45 qualitative studies on the work-family interface in academia, found that although academics valued the flexibility of their roles, which helped them manage work-family demands, they still experienced significant levels of work-family conflict. Johnston *et al.* (2022) similarly found that while flexibility in working hours, combined with organizational support, positively influenced work-life balance, it remained insufficient to offset the effects of unbounded work in academia.

The lack of clear boundaries within academic roles - spanning teaching, research, funding applications, and administrative tasks - continues to significantly undermine work-life balance for many academics ( Johnston *et al.*, 2022). Griffin (2022, p. 2190) introduces the concept of "work-work balance" to describe the challenge academics face in managing multiple, often conflicting, work demands. Griffin outlines four scenarios of imbalance: splitting time between different jobs, managing multiple projects, juggling various roles, and dealing with conflicting expectations within a single role. This imbalance not only affects academics' ability to meet work and personal life responsibilities but also reflects structural issues within higher education institutions and funding frameworks ( Griffin, 2022).

***Gendered boundary work*.** Gender inequalities further complicate the work-life balance landscape in academia. Research consistently identifies work-life balance as a major barrier to women’s career advancement within higher education institutions (HEIs) ( Beddoes & Pawley, 2014; Westoby *et al.*, 2021). Beddoes and Pawley (2014) found that STEM faculty often framed work-life balance challenges, long work hours, and unequal childcare responsibilities as personal choices rather than systemic issues, effectively “de-problematizing” structural inequalities and shifting responsibility away from institutions. Similarly, Westoby *et al.* (2021) identified six key themes in their review of gender inequalities in HEIs, including exclusion from networks, work-home balance difficulties, and everyday sexism. Both studies emphasize the persistence of biases and barriers for women in academia, calling for institutional reforms and cultural shifts to address these entrenched inequities.

Gendered divisions of labour at home are often mirrored in the workplace, where women disproportionately shoulder the burden of academic "care work"—both physical and emotional ( Berheide *et al.*, 2022; Docka-Filipek & Stone, 2021; Guarino & Borden, 2017). These patterns intensified during the pandemic, as women engaged in more service work at the expense of research productivity and career progression, activities that are typically more highly valued by institutions ( Berheide *et al.*, 2022; Docka-Filipek & Stone, 2021). Female faculty were also more likely to undertake relational service work, such as mentoring or responding to students' needs, compared to their male counterparts ( Berheide *et al.*, 2022). This type of emotional labour, though often invisible, diverts valuable time away from research—an activity central to promotion and tenure decisions—while also draining personal resources.

These gendered divisions extend to the management of work-family boundaries. Beigi *et al.* (2018), in their review of 45 qualitative studies on the work-family interface in academia, found distinct gendered patterns in boundary management. Beigi *et al.*’s (2018) review suggests that men generally preferred and were more successful in maintaining clear separations between work and family life (e.g., Damaske *et al.*, 2014; Reddick *et al.*, 2012, as cited in Beigi *et al.*, 2018). In contrast, women - particularly those with young children—were more likely to experience boundary-crossing, whether by choice or necessity (e.g., Heijstra & Rafnsdóttir, 2010, as cited in Beigi *et al.*, 2018). This reflects broader gendered differences in the strategies employed to navigate work-family roles, with women more frequently struggling to separate professional and personal demands in academic settings.

***Neoliberal management principles and work-nonwork boundaries*.** In addition to gender inequalities, the rise of neoliberal management practices within academia has exacerbated challenges in managing work-nonwork boundaries. Universities are increasingly adopting neoliberal principles, resulting in heightened managerialism and careerism ( Alvesson & Spicer, 2016; Billsberry *et al.*, 2023; Clarke & Knights, 2015; Fleming, 2023), alongside job instability, especially among early-career scholars ( Bozzon *et al.*, 2017; Bristow *et al.*, 2017). This shift, coupled with the growing emphasis on ‘performance’ metrics and career progression, has blurred the boundaries between work and personal life, with academics compelled to prioritize work-related activities and achievements over well-being ( Ylijoki, 2013). The persistent pressure to secure funding and meet performance guidelines often leads to longer work hours, encroaching on personal time and complicating efforts to establish sustainable work-life boundaries ( Hogan *et al.*, 2014).

Furthermore, the adoption of neoliberal principles in academia challenges core scholarly values such as intellectual freedom and collegiality ( Fleming & Harley, 2024). Collegiality, once valued for fostering a supportive academic environment, has become a mechanism for control, pressuring academics into performing unrecognized and uncompensated work to comply with institutional demands ( Fleming & Harley, 2024). Internalized pressures, particularly around publishing and securing research funding, contribute to growing insecurity, eroding both academic identity and research quality while complicating the work-nonwork interface ( Clarke & Knights, 2015; Knights & Clarke, 2014; Ylijoki, 2013).

The clash between traditional academic values and neoliberal principles has led to significant tension, alienation, and a sense of detachment from professional identity, prompting efforts to establish clearer boundaries in order to mitigate value dissonance ( Alvesson & Szkudlarek, 2021). In response to the intensified stress and alienation, some academics have engaged in various forms of resistance ( Contu, 2020; Kalfa *et al.*, 2018; Rintamäki & Alvesson, 2023). One notable example is the use of autoethnographic accounts, where researchers articulate their experiences of work-related stress within the fast-paced university environment and its impact on their personal lives (e.g., Cohen *et al.*, 2009; Izak *et al.*, 2023; Lupu, 2021). These accounts often reveal personal and sometimes difficult experiences related to mental, emotional, and physical health within academia (e.g., Boncori & Smith, 2019).

Such personal narratives offer insights into the intersection of personal hardship and professional life, underscoring the often unseen challenges academics experience ( Boncori & Smith, 2019; Contu, 2020; Kalfa *et al.*, 2018; Lupu, 2021). These accounts challenge existing taboos surrounding open discussions of mental health, well-being and loss in academia, as a way to preserve academic freedom as a core value but also to establish new standards of transparency and accountability concerning academic misconduct ( Boncori & Smith, 2019; Bunds, 2021; Smith & Ulus, 2020). By integrating discussions of work-nonwork boundaries into this discourse, these academics strive to maintain a coherent sense of identity and integrity amid the neoliberal landscape of higher education ( Clarke & Knights, 2015; Fitzmaurice, 2013; Kallio *et al.*, 2016).

Additionally, these narratives reflect a growing dissatisfaction with neoliberal management control models ( Billsberry *et al.*, 2023). By openly discussing physical and mental health issues ( Boncori & Smith, 2019; Contu, 2020; Kalfa *et al.*, 2018; Lupu, 2021), academics seek to reclaim autonomy over their work and personal lives. The blending of personal/private and professional/public experiences in academic literature represents an effort to establish more sustainable and coherent delineations between these realms.

***Internationalization and digitalization in academic research*.** The internationalization of academic research, coupled with the impacts of digitalization, has significantly transformed the landscape of academic work, leading to a profound blurring of boundaries between professional and personal life ( Collins *et al.*, 2022). With constant connectivity, academics can engage in work-related activities from virtually any location—be it at home, during travel, or elsewhere ( Pluut & Wonders, 2020). This flexibility means that traditional geographical and temporal constraints are no longer applicable; academics can attend conferences, teach students, and collaborate with colleagues globally at any time. However, this shift has also led to the erosion of established boundaries, making the distinction between work and non-work increasingly nebulous. Consequently, maintaining a balanced and sustainable work-life boundary has become a significant challenge for many scholars.

As research becomes more internationalized ( Altbach & Knight, 2007; Dachs *et al.*, 2024; Haley *et al.*, 2024), the conventional image of university academics as independent scholars with the autonomy to direct their own research ( Parker & Jary, 1995) appears increasingly misleading. In reality, academics are required to navigate heightened standards and expectations that arise not only from institutional hierarchies but also from global market demands ( Dachs *et al.*, 2024).

In this competitive global environment, research is increasingly regarded as a vital asset, driving economic growth and promoting human development ( Barrera, 2022). As a result, professors find themselves competing not just within their universities but against scholars from around the world. This competition is influenced by metrics such as university rankings and citation counts, often necessitating sacrifices of personal time in pursuit of academic success ( Johnston *et al.*, 2022).

While digitalization may offer the promise of increased flexibility in work arrangements ( Tims *et al.*, 2022), it simultaneously intensifies the pressure to produce research and engage with global colleagues. This expanded scope of responsibilities often intrudes upon personal time, complicating the work-life balance further ( Pasamar *et al.*, 2020). Additionally, geographic and economic disparities exacerbate these challenges, as working conditions for academics can vary significantly across different countries ( Bentley & Kyvik, 2012). For example, in nations where academic wages are lower, scholars may be compelled to hold multiple positions to sustain their livelihoods ( Griffin, 2022).

### The question of structural vs. individual-based understanding

The work-nonwork interface literature often examines how structural and individual factors influence individuals' navigation of work and nonwork boundaries ( Beigi *et al.*, 2018). While each perspective provides valuable insights, they frequently fail to address the interconnectedness between these factors. For example, individual traits like over-commitment are not isolated but can be amplified by structural conditions, such as high workloads or organizational cultures that promote excessive dedication.

To move beyond analyzing structural or individual factors in isolation, Tomkins ( 2017, p. 96) highlights that “context is inextricably interwoven with, and constitutive of the experience itself.” This perspective emphasizes the need to examine the ‘person-in-context,’ which requires understanding how individuals interpret and respond to the practices, expectations, and norms around them, including those shaped by institutions and society.

From a contextualist perspective ( Braun & Clarke, 2022), individuals and their environments are inseparably linked, with personal behaviors emerging from interactions with both structural and cultural contexts. Critical management scholars (e.g., Bristow *et al.*, 2017; Fleming & Harley, 2024) reinforce this view, arguing that organizational practices - such as performance metrics or norms prioritizing constant availability - not only influence individual behaviors but also actively shape and reinforce them.

Adopting this contextualist lens allows researchers to explore the mutually constitutive relationship between individuals and their environments. By recognizing the deep interconnection between structural and individual factors, this approach advances understanding of boundary dynamics and moves beyond the limitations of viewing these elements as separate and independent.

### The question of construct vs. experiential understanding

The literature on work-nonwork relationships often adopts a construct-led and correlational approach, focusing on established concepts such as conflict, balance, and boundaries. While these frameworks provide valuable insights, they tend to oversimplify the nuanced, context-specific experiences of individuals navigating boundary challenges ( Tomkins & Eatough, 2014). This approach frequently presents these concepts as static or universally applicable, failing to capture the fluid, dynamic, and multifaceted nature of individual experiences across diverse contexts.

Over-reliance on constructs in organizational studies has been criticized for creating overly broad or overly restrictive interpretations of phenomena. For instance, Alvesson and Blom (2022) critique the use of "hembig concepts" such as leadership and strategy, which can simultaneously encompass a wide range of ideas while offering limited explanatory precision. Tomkins and Eatough (2014) argue that such constructs impose rigid boundaries on understanding phenomena, restricting our ability to account for the complexity and variability inherent in real-world experiences.

This critique suggests that while concepts and constructs can serve as useful tools for organizing and analyzing phenomena, they should not overshadow the importance of understanding how individuals experience, interpret, and navigate work-nonwork relationships in specific contexts. By prioritizing lived experiences and embracing the variability of these dynamics, researchers can move beyond the limitations of constructs and develop a more comprehensive understanding of real-life challenges.

Moreover, mainstream research frequently neglects contributions from studies not explicitly focused on these specific work-nonwork interface concepts, despite their relevance to boundary dynamics. For example, as earlier discussed research by Boncori and Smith (2019), Contu (2020), Kalfa *et al.* (2018), and Lupu (2021) explores themes like identity, emotion, and structural inequalities, which directly touch upn how work-nonwork dilemmas are experienced and negotiated. By sidelining these perspectives, the field risks failing to capture the full complexity of work-nonwork boundaries as lived phenomena shaped by both structural conditions and individual agency.

## Discussion and conclusions

The review underscores the persistent challenges faced by academics as work-nonwork boundaries become increasingly blurred, shaped by the unique context of academic work. Academic working patterns contribute significantly to this blurring, encapsulated by the concept of "liquid liminality" ( Izak *et al.*, 2023). These challenges are particularly pronounced for women, who disproportionately engage in service and emotional labour - exacerbated during the pandemic - leading to reduced research productivity and stalled career progression ( Docka-Filipek & Stone, 2021; Guarino & Borden, 2017). By critically examining existing research, this review offers a more nuanced synthesis that accounts for the specific characteristics of academic work, such as unbounded work, perceived autonomy, and self-management. This perspective deepens the understanding of how work-nonwork boundaries in academia differ from those in other professions, shedding light on the unique complexities academics encounter in navigating the boundaries between their professional and personal lives.

Neoliberal pressures within academia, including performance metrics such as publication counts, citation rates, grant acquisition, and student satisfaction rates, exacerbate challenges in managing boundaries between work and personal life, often leading to overwork and poor work-life balance ( Rosa, 2022; Ylijoki, 2013). Additionally, the processes of internationalization and digitalization introduce further complexities. The rapid expansion of global academic networks and the increasing reliance on digital platforms have eroded traditional spatial and temporal boundaries. Academics are now expected to collaborate, teach, and conduct research across multiple time zones, which further blurs these distinctions ( Pluut & Wonders, 2020). The pressure to meet global standards, participate in international collaborations, and respond to work demands outside conventional hours intensifies this issue, adding another layer of difficulty in maintaining healthy boundaries ( Johnston *et al.*, 2022). Despite the stress and alienation experienced by many, some academics actively resist these trends by sharing personal narratives of their experiences, aiming to redefine boundaries and challenge the dominance of neoliberal values ( Boncori & Smith, 2019; Lupu, 2021).

### Problematizing the current research understanding and future directions

The review contextualizes work-nonwork boundaries within the academic profession by challenging the conventional frameworks that assume work-nonwork conflict or work-life balance approaches, which lack the understanding of blurred or dissolved boundaries between work and nonwork and often treat these boundaries as universally clear. While existing reviews ( Beigi *et al.*, 2018; Rosa, 2022) have provided valuable insights, they have been critiqued for simply accumulating knowledge without critically examining the underlying assumptions. These approaches tend to overlook the unique demands and structures of academic work, often emphasizing broad, abstract concepts such as "work-life balance", "work-nonwork conflict" or "flexible work" without fully considering why these boundaries are constructed and negotiated within academia. We recommend that future research transition from a concept-driven approach to one that is grounded in experiential and contextual realities. The current literature on work and nonwork often focuses too heavily on theoretical constructs such as conflict, balance, and boundaries, while overlooking the lived experiences of individuals. This oversight, especially common in quantitative studies, tends to oversimplify the complex realities of academics' work and nonwork lives.

Our review highlights a notable gap in mainstream research and literature regarding work and nonwork: the frequent neglect of studies where the work-nonwork interface is not the primary focus (e.g., Boncori & Smith, 2019; Contu, 2020; Kalfa *et al.*, 2018; Lupu, 2021). Although these studies offer valuable insights into work and nonwork experiences, they are often excluded from discussions about the "work-nonwork boundary." This exclusion limits the field's ability to develop a more nuanced understanding of these boundaries and their dynamic nature. By addressing this oversight, we can achieve a richer, multidimensional perspective on how individuals navigate work-nonwork boundaries in academia.

This review addresses a critical gap in the literature by focusing specifically on how academics, as a distinct professional group, experience and manage the boundaries between their work and personal lives. Despite the well-documented pressures of academic work, few reviews have fully explored why and how these pressures shape the construction and navigation of work-nonwork boundaries. This review, by reassessing and challenging current assumptions, provides a deeper understanding of the shared boundary challenges faced by academics. By adopting a problematizing approach ( Alvesson & Sandberg, 2020), this review contributes to a more context-sensitive exploration of work-nonwork boundaries in this demanding professional environment.

We suggest that future research would benefit from adopting a contextualist approach that recognizes the interconnectedness of individual and structural factors in shaping the boundaries between work and non-work. Although some studies have examined individual variations in boundary management and others have explored the professional work context, it is clear that boundary management is not an isolated experience; rather, it is deeply embedded in an individual’s social and professional environment. Contextualism emphasizes that individuals cannot be meaningfully understood without considering the contexts in which they live and work ( Braun & Clarke, 2022).

This principle is reflected in our review approach and findings, which suggest that boundary management is a co-constructed process shaped by both the individual’s choices and actions, as well as the external context in which they are situated. In essence, boundary management arises not only from personal decisions but also from a dynamic interaction between the individual and the surrounding social, organizational, and cultural factors.

In conclusion, this review critiques the traditional approach of viewing boundary negotiation as merely a problem to be solved, advocating instead for a more nuanced understanding of the fluid and context-dependent nature of these boundaries in academia. The challenges faced by academics—exacerbated by non-traditional work hours, gendered divisions of labor, and neoliberal pressures—underscore the need for a shift in perspective.

## Limitations

This review primarily draws on critical management literature to explore how the work context shapes academics' work-nonwork boundaries. While this approach offers valuable insights, it may inadvertently exclude perspectives from disciplines outside the social sciences. For instance, fields in STEM and the natural sciences often have distinct work cultures and expectations that may shape boundary experiences differently. Future reviews should consider incorporating a broader range of disciplines to capture diverse academic experiences and provide a more comprehensive understanding of how work-nonwork boundaries are negotiated across various fields. One limitation of this review relates to the differences among various types of institutions. Research institutions can differ significantly from those that focus exclusively on teaching. These distinctions can be further affected by factors such as funding sources (public versus private), the size of the faculty and student populations, and other characteristics specific to each institution.

## Ethical approval and consent

No ethical approval or consent required.

## Data Availability

No data are associated with this article. No underlying data are associated with this article. Zenodo : **Work-Nonwork boundaries in academia: A problematizing review** (the DOI 10.5281/zenodo.14006365) **The project contains the following data** ; Table 2-Level 2-Readings License CC BY 1.0 universal
