# Supplementary material for: Work-Nonwork boundaries in academia: A problematizing review
Source: Open Res Eur. 2025 Feb 5;4:245. Originally published 2024 Nov 7. [Version 2] doi: 10.12688/openreseurope.18573.2 (PMC11826078; doi:10.12688/openreseurope.18573.2)
Supplement: Peer review history

# Reviewer report: Reviewer response for version 2

**DOI:** 10.21956/openreseurope.21128.r50792

**Published:** 2025-03-21

**Authors:** Anna Carreri

**Affiliations:** 1 Università di Verona, Hasselt University, Verona, Italy

Dear authors,

 thanks for your responses.

 I think your work has now improved a lot. Well done!

Are all factual statements correct and adequately supported by citations?

Yes

Is the review written in accessible language?

Yes

Are the conclusions drawn appropriate in the context of the current research literature?

Partly

Is the topic of the review discussed comprehensively in the context of the current literature?

Partly

Reviewer Expertise:

work-life interface; work and gender inequalities; intersectionality; new forms of work; meaningful work; alienation; flexible work; academic work; knowledge work; qualitative methods

I confirm that I have read this submission and believe that I have an appropriate level of expertise to confirm that it is of an acceptable scientific standard.

# Reviewer report: Reviewer response for version 2

**DOI:** 10.21956/openreseurope.21128.r50794

**Published:** 2025-02-13

**Authors:** Gail Kinman

**Affiliations:** 1 University of Nottingham, Nottingham, England, UK

Dear Jūratė and Aleksandra, Thank you for such a detail response to my comments - I am glad you found them helpful. I think the article is very much strengthened following the revisions based on the three reviewers' comments.  I believe it makes a strong, novel contribution to the literature.  My only comment relates to the meaning of the term 'seminal' - providing a definition or some criteria for this would be helpful, but I appreciate this may be challenging.

Are all factual statements correct and adequately supported by citations?

Partly

Is the review written in accessible language?

Yes

Are the conclusions drawn appropriate in the context of the current research literature?

Partly

Is the topic of the review discussed comprehensively in the context of the current literature?

Partly

Reviewer Expertise:

I have longstanding expertise in the topic and the issues discussed in the article and have published widely in the area.

I confirm that I have read this submission and believe that I have an appropriate level of expertise to confirm that it is of an acceptable scientific standard.

# Reviewer report: Reviewer response for version 2

**DOI:** 10.21956/openreseurope.21128.r50793

**Published:** 2025-02-06

**Authors:** Joel B Tan

**Affiliations:** 1 University of Mindanao, Davao City, Philippines

I am ok with the revised article. Thank you.

Are all factual statements correct and adequately supported by citations?

Yes

Is the review written in accessible language?

Yes

Are the conclusions drawn appropriate in the context of the current research literature?

Partly

Is the topic of the review discussed comprehensively in the context of the current literature?

Yes

Reviewer Expertise:

Business Management, Human Capital, Quality Management

I confirm that I have read this submission and believe that I have an appropriate level of expertise to confirm that it is of an acceptable scientific standard.

# Reviewer report: Reviewer response for version 1

**DOI:** 10.21956/openreseurope.20087.r47360

**Published:** 2025-01-11

**Authors:** Anna Carreri

**Affiliations:** 1 Università di Verona, Hasselt University, Verona, Italy

This literature review (41 readings) focuses on the specific dynamics of work-nonwork boundaries characterizing academic settings.  The objective is to advance the conversation beyond traditional approaches/concepts of work-life ‘balance’ and work-life ‘conflict’, and to emphasize how the specific work context influences the construction and negotiation of boundaries. Thank you for the opportunity to read this interesting work on a topic of such theoretical importance.

 I think the objective of the literature review is noteworthy. I am drawn to the concept of boundary as it is one that potentially better captures dynamic processes (although only partially) and I myself have used it several times in my studies on the interface between work and life, also with specific reference to academic work during Covid-19, and more generally in knowledge work. Indeed, it is a concept that allows us to grasp the links with organizational contexts and the dimensions of gender, generation, etc., allowing us, in part, to move away from the problematic assumptions about individualism and power neutrality that characterize most of WLB literature, an aspect that is surprisingly not problematised in this review.

 The problematising review approach is interesting in my opinion in the way it is described. However, I find the choice of books in the third stage of the selection process questionable (a bit unfocused and discretionary). At least, it should be better argued, I think. Moreover, what was actually done is not explained. *How* were the texts read and analysed: which questions? which steps? which codes, if any? I suggest expanding this part and providing more information on the analysis process.

 More importantly, I think that the objectives of the problematizing review approach have not been achieved. The article offers a content review in which the main findings are grouped within a few themes in this current version. There is no critical reasoning on the dominant paradigms, conceptual assumptions, and conventions within the work-nonwork literature. We learn what we already know, i.e., that academic work is characterized by blurred boundaries that undermine work-life balance for many academics (importantly, some more than others, e.g., not only women but also women with children and even more so young and precarious women with children - intersectional reading should also be included!). This reading of the texts problematizes little and does not explore alternative perspectives as stated in the method's description.

 Finally, the review does not focus on the concept of boundary (or micro-transitions), but more generally on the work-life interface of academics. There is a lack of reasoned articulation of the concept of boundary based on the readings included in the review (also because several of the included studies do not use the concept of boundary - an aspect that I think needs to be argued effectively).

 I believe that the work can be useful in its objectives and potentially of great interest, but it needs more analytical-interpretive effort. I hope that my comments will be helpful to you in developing the review.

Are all factual statements correct and adequately supported by citations?

Yes

Is the review written in accessible language?

Yes

Are the conclusions drawn appropriate in the context of the current research literature?

Partly

Is the topic of the review discussed comprehensively in the context of the current literature?

Partly

Reviewer Expertise:

work-life interface; work and gender inequalities; intersectionality; new forms of work; meaningful work; alienation; flexible work; academic work; knowledge work; qualitative methods

I confirm that I have read this submission and believe that I have an appropriate level of expertise to confirm that it is of an acceptable scientific standard, however I have significant reservations, as outlined above.

# Response

**Published:** 2025-02-03

**Authors:** Jūratė Čingienė

**Affiliations:** SMK University of Applied Social Sciences, Vilnius, Vilnius County, Lithuania

Dear Prof Anna Carreri,

 Thank you for your detailed and thoughtful feedback on our article. We genuinely appreciate your insights and the effort you put into engaging with our work. Your comments have been important in helping us improve the clarity and focus of the paper, which we have now prepared in version 2.

 Based on your feedback, we have made several significant changes. First, we improved the methodology section by providing a more detailed description of the analysis and the review process. More importantly, we critically evaluated the dominant paradigms and conceptual assumptions within the review to better align with the goals of the problematization review method. We propose an alternative approach that emphasizes both experiential and contextual perspectives.

 Regarding the concept of boundaries, we recognize that we did not exclusively consider studies that are specifically dedicated to this topic as we included research on work-life balance and conflict. We did this because these areas also address boundary issues.

 We now highlight that much of the existing research tends to focus on concepts rather than seeking to develop an experiential understanding, which we also problematize in the revised version.

 Thank you once again for your invaluable insights and for sharing information about your research.

 Best regards,

 Jūratė and Aleksandra

# Reviewer report: Reviewer response for version 1

**DOI:** 10.21956/openreseurope.20087.r47368

**Published:** 2024-12-13

**Authors:** Joel B Tan

**Affiliations:** 1 University of Mindanao, Davao City, Philippines

The study highlights how academic work's unique characteristics such as unbounded tasks, perceived autonomy, and neoliberal pressures intensify the blurring of work-nonwork boundaries. The conclusion could explore how the findings could be translated into action plans and could influence broader discussions about professional boundaries in other knowledge-based or high-autonomy fields.

 While the study has pointed out the contributory effects of digitalization and globalization in shaping work-nonwork boundaries, it could also explore the influence of cultural diversity and institutional dynamics in boundary management. Another interesting angle for discussion is the role of intersectional identities (e.g., race, socioeconomic status, or disability) intersect with gender in shaping work-nonwork boundaries.

 On another note, while the review challenges conventional frameworks, it could further articulate how its problematizing approach advances theoretical understanding for studying work-nonwork boundaries across cultures, generations, and communities. 

 Ending with a clear and motivating call to action for academics, administrators, or policymakers would leave a lasting impression and reinforce the importance of addressing the challenges of work-nonwork boundaries in academia.

Are all factual statements correct and adequately supported by citations?

Yes

Is the review written in accessible language?

Yes

Are the conclusions drawn appropriate in the context of the current research literature?

Partly

Is the topic of the review discussed comprehensively in the context of the current literature?

Yes

Reviewer Expertise:

Business Management, Human Capital, Quality Management

I confirm that I have read this submission and believe that I have an appropriate level of expertise to confirm that it is of an acceptable scientific standard.

# Response

**Published:** 2025-02-03

**Authors:** Jūratė Čingienė

**Affiliations:** SMK University of Applied Social Sciences, Vilnius, Vilnius County, Lithuania

Dear Prof. Joel B. Tan,

 Thank you very much for your thoughtful and insightful feedback.

 Your suggestions have been instrumental in improving our article’s version 2, and we have made revisions based on your recommendations, as outlined below. We recognize the need to translate our findings into actionable recommendations and to better connect them to the broader discussion. To address this, we have revised the Discussion and Conclusions section to include specific recommendations for research on work-nonwork boundaries. Additionally, we have strengthened the conclusions by outlining clearer directions for future research. We also acknowledge that our initial focus on digitalization and globalization did not adequately consider the role of cultural diversity and institutional factors in shaping boundary management. In the Limitations section, we now explicitly acknowledge these variations, which were not fully addressed in our study. Furthermore, we have included recommendations for future research to explore institutional differences and cultural diversity to gain a better understanding of their influence. Additionally, we have expanded our discussion to highlight how this study contributes to the theoretical understanding of work-nonwork boundaries. We particularly emphasize the importance of experiential and contextual research methods in advancing this field. We sincerely appreciate your valuable contributions and believe that these revisions will enhance the comprehensiveness and impact of our study. Please let us know if there are any other aspects you would like us to address.

 Best regards, 

 Jūratė and Aleksandra

# Reviewer report: Reviewer response for version 1

**DOI:** 10.21956/openreseurope.20087.r46827

**Published:** 2024-12-02

**Authors:** Gail Kinman

**Affiliations:** 1 University of Nottingham, Nottingham, England, UK

Research shows that academic employees often experience difficulty in maintaining clear boundaries between ‘work’ and ‘non-work’, which can result in conflicts that impact their wellbeing and job performance. The unique nature of academic work and the institutions that employ them means that an occupationally specific approach has more potential to gain insight into academics’ boundary management experiences. Such insights can help inform interventions to help individuals and institutions enhance boundary management that, in turn, will support their wellbeing and productivity, as well as their work-life balance and quality of life.  For these reasons, I very much welcome the contextual approach taken in this review.

 This article considers the boundary challenges faced by academic employees, examining how the idiosyncratic demands and characteristics of academic work contribute to the blurring of boundaries between work and non-work. The article is generally well written and the authors clearly have an excellent, in-depth knowledge of the relevant literature.  I enjoyed reading this article – it raises some very interesting and relevant issues. Nonetheless, while I can appreciate the need to focus on boundary management, I am not convinced that this approach has fulfilled the aim to provide novel insights and a more nuanced understanding of academics’ experiences of negotiating (balancing/integrating etc) work and non-work life.

 The problematizing approach to reviewing literature is new to me but seems an interesting and novel way to gain insight. I appreciate the need for selective reading, but I need more information on the basis on which the 14 ‘seminal’ studies, the 27 key texts and the 3 books were selected. What makes a study seminal?  Is it a high number of citations?  Were the studies selected for their breadth, as they have very different aims and use diverse methodologies (including meta-synthesis, autoethnography and commentary, as well as structural equation modelling). The approach to sampling taken in these studies is diverse with a high proportion of these studies focusing on gender, particularly the experiences of women academics. The studies were also published over quite a wide timescale. I am not suggesting that this approach is problematic, but the process followed needs to be more transparent. Indeed, there are many relevant studies that could have been included that were not with some focusing more explicitly on boundaries in academic samples. A rationale for the choice of books is also needed – Nippert Eng is a classic in the field, but there are many other ‘general’ texts to choose from. How the information arising from these studies was extracted and integrated also needs further explanation to somebody who isn’t an expert in the process.

 The authors have extracted some key messages from the broad range of studies. Nonetheless, given the aims of the article, I feel that more focus is needed on bringing out what is new, or at least more focus on the type of questions that we need to ask in future studies to gain further insight.  Many previous studies have highlighted the gendered nature of the academy and the major challenges faced by women in managing boundaries between work and personal life. Other studies have identified the difficulties that academics experienced during the pandemic, and how the flexible and ‘unbounded’ nature of academic work and neoliberal pressures can intensify the impact of academic workloads and make boundary management more challenging.

 While structural factors, such as job demands, lack of support, digitalisation etc., are undoubtedly important in influencing boundary management, I feel a more systemic approach is needed. It is particularly important to consider the individual behaviours and actions academics use to navigate these boundaries that make them more or less permeable and flexible. To gain insight into boundary management, we also need to consider individual attitudes and orientations towards academic work.  My own research, along with that of others, highlights the impact of over-involvement and over-commitment to the work role. These factors can interact with heavy workload demands within organisational cultures that not only overlook, but often reward excessive dedication to work.  This will undoubtedly have an impact on boundary management. Moreover, the flexibility often found in academic work, when combined with work overload, can lead to enabled intensification which can hinder effective boundary management, particularly for academics working at home.

 I think the authors approach is interesting with potential to gain new insights.  I hope they find my comments and suggestions useful.

Are all factual statements correct and adequately supported by citations?

Partly

Is the review written in accessible language?

Yes

Are the conclusions drawn appropriate in the context of the current research literature?

Partly

Is the topic of the review discussed comprehensively in the context of the current literature?

Partly

Reviewer Expertise:

I have longstanding expertise in the topic and the issues discussed in the article and have published widely in the area.

I confirm that I have read this submission and believe that I have an appropriate level of expertise to state that I do not consider it to be of an acceptable scientific standard, for reasons outlined above.

# Response

**Published:** 2025-01-28

**Authors:** Jūratė Čingienė

**Affiliations:** SMK University of Applied Social Sciences, Vilnius, Vilnius County, Lithuania

Dear Prof Gail Kinman,

 Thank you for your detailed and thoughtful feedback on our article. We appreciate the time and care you have taken to engage with our work, and your comments have been invaluable in helping us improve its clarity and focus.

 Below, we outline our understanding of your points and the changes we have made in response, which are in the process of publishing as version 2.

**Clarity on the selection process for texts** -

 You highlighted the need for greater transparency in how and why the texts were selected and the criteria for selected works. We appreciate this observation and have revised the Methods section to provide a more detailed rationale for our selection process. 

**Transparency in the integration process** -

 We understand that the method of synthesizing insights across studies needed clearer articulation, particularly for readers less familiar with problematizing review approach. To address this, a new section “Review process” elaborates on how the review process was conducted.  Novel insights and future directions While the article explores the challenges of boundary management, we understand that it may not be clear how it offers novel insights or a more nuanced understanding. We have now refined the findings and discussion to emphasize the contributions of our article.

 Specifically, in the discussion and conclusions sections we now clearly highlight the key needs for future research:

 1) a shift from a concept-driven approach to an experiential-driven one and

 2) contextually grounded understanding. 

**Structural vs. Individual Factors** -

 We understand that while the article emphasizes structural influences (e.g., job demands, digitalization, neoliberal pressures), there might be a need for a more systemic approach that also considers individual behaviours, attitudes, and orientations toward work. Our suggested approach is now discussed in more detail in the Findings and Discussion sections. 

 We are grateful for your constructive feedback, which has significantly strengthened our work.

 The article now provides a clearer rationale for the selection of studies, a more transparent methodological approach, and an enhanced focus on future study directions.

 We believe these revisions make a stronger case for the article’s contributions and offer more actionable insights for improving academic boundary management.

 Thank you again for your thoughtful input - it has been instrumental in improving the depth and clarity of the article.

 Kind regards,

 Jūratė and Aleksandra
